# Zika Virus NS3 Drives the Assembly of a Replication Compartment-like Structure That Exerts the Structural and Physiological Functions of the Viral Replication Compartment

**DOI:** 10.3390/v18080834

**Published:** 2026-07-29

**Authors:** Tania Sultana, Chunfeng Zheng, Jenna Jones, Nina Zamani, Maria Pilar Toledo, Garret M. Morton, Yue J. Wang, Timothy L. Megraw

**Affiliations:** 1 Department of Biomedical Sciences, College of Medicine, Florida State University, Tallahassee, FL 32306, USA; ts21m@fsu.edu (T.S.); chunfeng.zheng@med.fsu.edu (C.Z.); nina.zamani@med.fsu.edu (N.Z.); pilar.toledo@med.fsu.edu (M.P.T.); julia.wang@med.fsu.edu (Y.J.W.); 2 Department of Biological Science, Florida State University, Tallahassee, FL 32306, USA

**Keywords:** Zika virus, NS3, replication compartment, replication compartment-like structure, orthoflavivirus, helicase, UPR, unfolded protein response, CHOP, PERK

## Abstract

Zika virus (ZIKV) is a mosquito-transmitted orthoflavivirus that caused an epidemic in 2015–2016 in the Americas and raised serious global health concerns due to its association with congenital brain anomalies when infections occur during pregnancy. Various viruses can form compartments within the cell to facilitate viral replication and assembly, referred to as viroplasms, replication organelles, or virus factories depending on the type of virus. ZIKV assembles virus particles in virus-generated compartments adjacent to the nucleus, referred to here as a replication compartment (RC), which is formed by remodeling the host cell endoplasmic reticulum (ER). How the viral proteins control RC assembly remains unknown. Here we show that the ZIKV non-structural protein 3 (NS3), a dual-function protease and RNA helicase, is sufficient to drive the assembly of a replication compartment-like structure (RCLS) in human cells. While sufficient to generate the RCLS, NS3 is less efficient in several aspects compared to ZIKV-induced RC assembly. Nonetheless, the RCLS is similar to the ZIKV RC in its assembly at the nuclear periphery, its recruitment of ER, association with the Golgi and centrosome, and the arrangement of microtubules at its surface. Moreover, NS3 expression results in activation of the unfolded protein response (UPR), but attenuates expression of the downstream transcription factor CHOP, mirroring the manipulation of the different aspects of the UPR by ZIKV infection. We further show that the helicase domain and not the protease domain is required for optimal RCLS formation and organelle recruitment, yet each domain affects different control over the UPR. Overall, these findings advance our understanding of the mechanism of RC assembly by ZIKV, its involvement in hijacking the UPR, and the central role of NS3 in the process.

## 1. Introduction

The epidemic outbreak of the Zika virus (ZIKV) in 2015–2016 in the Americas raised serious health concerns worldwide due to the detrimental effects on fetal brain development and associated Guillain-Barré syndrome in adults [1]. ZIKV RNA has been detected in the placental and fetal brain tissues of pregnant mothers upon occurrence of infection. Moreover, ZIKV can infect neurons and neuronal progenitor cells, leading to microcephaly and other brain developmental defects [2,3].

ZIKV is a mosquito-transmitted orthoflavivirus related to Dengue virus (DENV), yellow fever virus (YFV), West Nile virus, and Japanese encephalitis virus (JEV) [4,5,6]. The ZIKV genome is a positive-sense single-stranded RNA of approximately 11,000 bases that encodes three structural proteins (capsid C, pre-membrane prM, and envelope E) and seven non-structural (NS) proteins (NS1, NS2A, NS2B, NS3, NS4A, NS4B, and NS5) expressed as a single polyprotein that is processed by host and viral proteases into the 10 individual viral proteins (Figure S1A) [7].

Many viruses, including ZIKV, organize specialized intracellular microenvironments within the host cell from viral and host proteins, and by reorganizing host organelles to replicate their genomes and assemble virus particles. These structures, generally referred to as replication compartments (RCs), exist in varied forms known as replication organelles, replication complexes, or virus factories [8,9,10,11,12,13]. RC organization can vary among different viruses. Although the size, shape, and composition of the RC varies depending on the types of virus and host cells, orthoflavivirus RCs are derived from the endoplasmic reticulum (ER) and include additional ER-derived features such as convoluted membranes and virus bags [14,15,16,17,18]. ZIKV replication at the ER activates the unfolded protein response (UPR) [19,20], which might serve to facilitate viral replication, but ZIKV infection also inhibits expression of UPR elicitor CHOP. CHOP is a transcription factor expressed in the nucleus upon UPR. Under prolonged ER stress, CHOP activates genes that promote apoptosis [21].

We previously showed that during ZIKV infection the RC is large, has a toroidal shape, and has the centrosome positioned within the core [22]. In the absence of centrosomes, the RC assembles but is spherical [22]. The RC recruits ER and Golgi and organizes microtubules (MTs) on its surface [22]. Here, in our pursuit of understanding the roles of individual ZIKV proteins in RC formation, we discovered a key role for the ZIKV NS3 protein. We found that ZIKV NS3 drives the assembly of a similar structure, a ‘replication compartment-like structure’ or RCLS, and similarly triggers the UPR through PKR-like endoplasmic reticulum kinase (PERK) activation while subverting later steps in the UPR by inhibiting CHOP. ZIKV NS3 is a dual-domain protein with an N-terminal trypsin-like serine protease domain and a C-terminal helicase domain [23,24]. The NS3 protease processes the ZIKV polyprotein but also cleaves key host proteins involved in the innate immune response [25]. The NS3 helicase utilizes the chemical energy derived from ATP hydrolysis to unwind double-stranded RNA during replication [26] and contains functional motifs found in RNA helicases, including Walker A and B motifs for ATP binding and hydrolysis, and a separate RNA binding domain [27]. We show that the compact organization of the RCLS, its recruitment of ER, integrity of centrosomes, localization of Golgi, organization of MTs, and manipulation of the UPR are impacted differentially by mutations affecting the NS3 helicase and the protease.

## 2. Materials and Methods

### 2.1. Cell Culture

Cells from the U-251 cell line derivative SNB-19 (Charles River Laboratories, Inc. Worcester, MA, USA, under contract of the Biological Testing Branch of the National Cancer Institute) were used for most virus infections and plasmid transfections. We also used HEK293T cells (ATCC, Manassas, VA, USA, cat# CRL-3216) for ZIKV infection and HeLa cells (a kind gift from Raed M. Rizkallah, assistant professor, Biomedical Science, FSU) for transfections and subsequent protein expression analysis via Western blot. SNB-19 cells were cultured and maintained in RPMI medium (Cytiva-HyClone, Logan, UT, USA, cat# SH30027.02) and HEK293T and HeLa cells on DMEM medium (Corning, Corning, NY, USA, cat# 10-0170-CV) on standard cell culture dishes (VWR International, Radnor, PA, USA, cat# 10062-890). Virus stock production was carried out using Vero E6 cells (ATCC, cat# CRL-1586) cultured in DMEM media. All media were supplemented with 10% fetal bovine serum (FBS) (Avantor Seradigm, Radnor, PA, USA, cat# 97068-85) and 1% penicillin-streptomycin (Corning, cat# 30-002-CI) and maintained in a humidified environment with 5% CO_2_ at 37 °C. To elicit ER stress and activate the UPR, cells were treated with 1 µM thapsigargin for 4 h [28].

### 2.2. Plasmids and Molecular Cloning

We employed Gateway cloning (Invitrogen, Carlsbad, CA, USA) to create entry clones in pENTR (Invitrogen, cat# 45-0218) and subsequently subcloned the inserts NS3 (ZIKV MR766; 1-617 aa), NS3-protease (ZIKV MR766; 1-167 aa), and NS3-helicase (ZIKV MR766; 168-617 aa) into the destination vector, pSG5-FLAG (a gift from Eric C. Johannsen [29], originally from Hatzivassiliou et al. [30]), by recombination using LR Clonase (Invitrogen, cat# 11791-020). We constructed pDEST53-GFP-MRZIKV-NS3 (GFP-NS3) by using the destination vector pDEST53-GFP (Invitrogen, cat# 12288015). The fusion constructs NS2B–NS3 and YFV-NS3 were amplified through PCR using PAGFP-NS2BNS3 (Addgene, Watertown, MA, USA, cat# 195755) and YFVdelNS1-Cre (Addgene, cat# 179952) respectively and were subsequently cloned in pSG5-FLAG. pULTRA-Chilli-PRVABC59-NS3 (PR-NS3) construct was generously provided by Dr. Hengli Tang (Florida State University). All other Flag-tagged ZIKV proteins were kindly gifted by Dr. Tang’s lab, originally from Hongjun Song’s lab [31]. The primers for cloning NS3 constructs are listed in Table S1.

### 2.3. Transfection

Cells were plated for ~24 h (h) in 12-well plates (Greiner, Monroe, NC, USA, cat# 665180) containing sterile 18 mm circular coverslips (Electron Microscopy Sciences, Hatfield, PA, USA, cat# 72222-01). Cells at 70–80% confluency were transfected using 1 µg of plasmid for 24 h for the detection of early ER recruitment and frequency of RCLS formation, whereas all other experiments were performed 48 h post-transfection. Lipofectamine 3000 reagent (Invitrogen, cat# L3000015) was used following the manufacturer’s protocol. After transfection, the media were removed and cells were fixed with 100% methanol (VWR International, cat# BDH2018-1GLP) at −20 °C for 10 min followed by three phosphate-buffered saline (PBS) washes. Non-transfected cells were used as control.

### 2.4. Virus Stock Production and Infection

We utilized the following virus strains: ZIKV-MR766 (MR-ZIKV; African lineage) (ZeptoMetrix, Franklin, MA, USA, cat# 0810521CF), ZIKV-PRVABC59 (PR-ZIKV; Asian-lineage Puerto Rican strain) (ATCC, cat# VR-1843), and YFV-17D-204 (YFV) (kindly provided by Dr. Hengli Tang). Virus stocks were prepared as described previously [32]. In brief, we infected Vero E6 cells with virus stock diluted in the culture medium at a multiplicity of infection (MOI) of 0.01 and allowed them to incubate with the cells for 2 h. The infection medium was replaced with fresh media, and the supernatant was harvested 72–96 h post-infection when a significant cytopathic effect was observed. The supernatants were centrifuged at 1000× *g* for 10 min, filtered through a 0.45-micron filter (VWR International, cat# 76479-028), aliquoted and stored at −80 °C.

SNB-19 cells were infected with MR-ZIKV, PR-ZIKV, and YFV, and HEK293T cells with MR-ZIKV, all at an MOI of 1.0 in culture medium and maintained for 24 h. Cell culture media were used for mock infection.

### 2.5. Site-Directed Mutagenesis

Mutations targeting the protease and helicase domains of MR-NS3 were introduced into the pSG5-FLAG-NS3 template using the QuikChange II site-directed mutagenesis kit (Agilent, Cedar Creek, TX, USA, cat# 200523-5), following the manufacturer’s protocol. Subsequently, all mutations were confirmed by DNA sequencing. Table S1 lists the sequences of the primers used for site-directed mutagenesis.

### 2.6. Western Blotting

Cells were seeded in 6-well plates (Greiner, cat# 657160) followed by transfection. SNB-19 cells were used for MR-NS3 mutants and HeLa cells for all other plasmid expressions. After 24 h, cells were washed with cold PBS, harvested, and lysed in SDS-PAGE loading dye. The lysates were boiled at 95 °C for 5 min and centrifuged at 4 °C for 5 min before loading onto SDS-PAGE gels. Protein bands were transferred to nitrocellulose membranes (VWR International, cat# 28298-020) which were blocked with 5% non-fat milk in Tris-Buffered Saline with Tween 20 (TBST). Following washing with TBST three times, membranes were incubated with primary antibodies overnight at 4 °C with gentle agitation and then with secondary antibodies for 2 h at room temperature (RT). Protein bands were visualized using the Odyssey CLx (LiCor Bioscience, Lincoln, NE, USA, cat# 9140) imaging system and analyzed using Image Studio Ver 5.2 (LiCor Biosciences).

### 2.7. Immunofluorescent (IF) Staining and Microscopy

For IF staining, methanol-fixed cells were treated with primary antibodies in PBS containing 0.1% saponin (Sigma-Aldrich, St. Louis, MO, USA, cat# SAE0073-10G) and 5 mg/mL bovine serum albumin (Boehringer Mannheim Corp., Indianapolis, IN, USA, cat# 100-021) for 2 h at RT or overnight at 4 °C, washed three times for 5 min in PBS, and then incubated with secondary antibodies for 2 h at RT. The wells were washed three times for 5 min with PBS and then mounted on slides (Fisher Scientific, Waltham, MA, USA, cat# 22-034486) in mounting medium [33].

Slides were imaged on a Nikon AX confocal microscope (Nikon, Melville, NY, USA) with a 60× NA 1.49 oil immersion objective using NIS-elements (Nikon) software (Version 5.42.04). All figures were composed using Adobe Illustrator (version 25.3.1).

### 2.8. Antibodies

The following primary antibodies were used for staining: rabbit polyclonal anti-ZIKV-NS3 (GeneTex, Irvine, CA, USA, cat# GTX133309, RRID: AB_2756864, 1:500 for IF, 1:10,000 for WB), chicken monoclonal anti-FLAG (Exalpha Biologicals Inc., Newark, CA, USA, cat# AFLAG, 1:500 for IF), rat monoclonal anti-FLAG (Agilent, cat# 200473, RRID: AB_10596510, 1:1000 for IF, 1:10,000 for WB), mouse monoclonal anti-FLAG (Sigma-Aldrich, St. Louis, MO, USA, cat# F3165, RRID: AB_259529, 1:1000 for IF, 1:10,000 for WB), rabbit polyclonal anti-ZIKV envelope protein (Kerafast, Newark, CA, USA, cat# EFS001, 1:500 for IF), mouse monoclonal anti-orthoflavivirus group antigen (envelope) (EMD Millipore, Burlington, MA, USA, clone D1-4G2-4-15, cat# MAB10216, RRID: AB_827205, 1:1000 for IF), rabbit polyclonal anti-ZIKV NS2B (GeneTex, cat# GTX133308, RRID: AB_2715494, 1:1000 for IF, 1:10,000 for WB), mouse monoclonal anti-KDEL (clone 10C3, Enzo Life Sciences, Farmingdale, NY, USA, cat# ADI-SPA-827, RRID: AB_2039327, 1:500 for IF), rabbit monoclonal anti-calnexin (Cell Signaling Technology, Danvers, MA, USA, C5C9, cat# 2679S, RRID: AB_2228381, 1:1000 for IF), and rabbit monoclonal anti-GM130 (Cell Signaling, D6B1, cat# 12480, RRID: AB_2797933 1:3000 for IF), mouse monoclonal anti-αtubulin (DM1A, Sigma-Aldrich, cat# T9026, RRID: AB_477593, 1:1000 for IF, 1:20,000 for WB), chicken anti-GFP (Antibodies Inc, Davis, CA, USA, cat# GFP-1020, RRID: AB_10000240, 1:500 for IF, 1:10,000 for WB), rabbit anti-GAPDH (Genetex, cat# GTX100118, RRID: AB_1080976, 1:10,000 for WB), rabbit polyclonal anti-CEP192 (Bethyl labs, Montgomery, TX, USA, cat# A302-324A, RRID: AB_1850234, 1:1000 for IF), and mouse monoclonal anti-GCP2 (Clone 01, a gift from Pavel Dráber, 1:500 for IF) [34], mouse anti-CHOP (Cell signaling, cat#2895, RRID: AB_2089254, 1:1000 for IF), and rabbit anti-pPERK (Abcam, Waltham, MA, USA, cat# ab192591, RRID: AB_991721, 1:1000 for IF).

We used goat anti-mouse (LI-COR, Lincoln, NE, USA, IRDye 800CW, cat# 926-32210, RRID: AB_621842 and IRDye 680RD cat# 926-68070, RRID: AB_10956588), goat anti-rabbit (LI-COR, IRDye 800CW, cat# 926-32211, RRID: AB_621843 and IRDye 680RD, cat# 925-68071, RRID: AB_2721181), and donkey anti-chicken (LI-COR, IRDye 800CW, cat# 926-32218, RRID: AB_1850023) 1:20,000 for WB and Alexa Fluor 488, 568, and 647 conjugated goat secondary antibodies (Invitrogen, 1:1000) for IF staining. Nuclei were stained using DAPI (Invitrogen, cat# D1306, 1 µg/mL for IF).

### 2.9. Image Analysis

Confocal images were analyzed using ImageJ (Fiji version 1.54p) [35]. To measure the mean fluorescence intensity of ER, pPERK, CHOP, and RCLS circularity, regions of interest (ROIs) were drawn around the appropriate compartment. Measurements were performed across relevant channels after enabling ‘mean gray value’ and ‘circularity’ in Analyze > Set Measurements. Background intensity was subtracted from raw values.

For colocalization analysis (Golgi or Acetylated tubulin with NS3), channels were split, the DAPI channel removed, and thresholds manually set (Golgi: 157/100; Acetylated tubulin: 102/100). The colocalization score was then calculated using the JaCoP plugin. The abundance of centrosomal proteins in RCLSs, as well as the frequency of RCLS formation, was determined through manual counting of immunostained slides. All quantifications were performed on 25–35 images per condition, obtained from a minimum of three independent experiments.

### 2.10. Statistical Analysis

The results in all graphs are presented as the mean ± standard deviation and analyzed by GraphPad Prism for Windows (GraphPad Software, version 10.0.0, Boston, MA, USA). Statistical analysis included one-way ANOVA followed by Dunnett’s post hoc test for multiple comparisons, or an unpaired Student’s *t*-test was carried out where appropriate to determine the differences in means between experiments, with a probability (*p* value) level of * *p* <  0.05, ** *p* <  0.01, *** *p* <  0.001, and **** *p* <  0.0001 considered increasingly significant, respectively.

## 3. Results

### 3.1. ZIKV NS3 Is Sufficient to Form a Replication Compartment-like Structure (RCLS)

**Figure 1:**
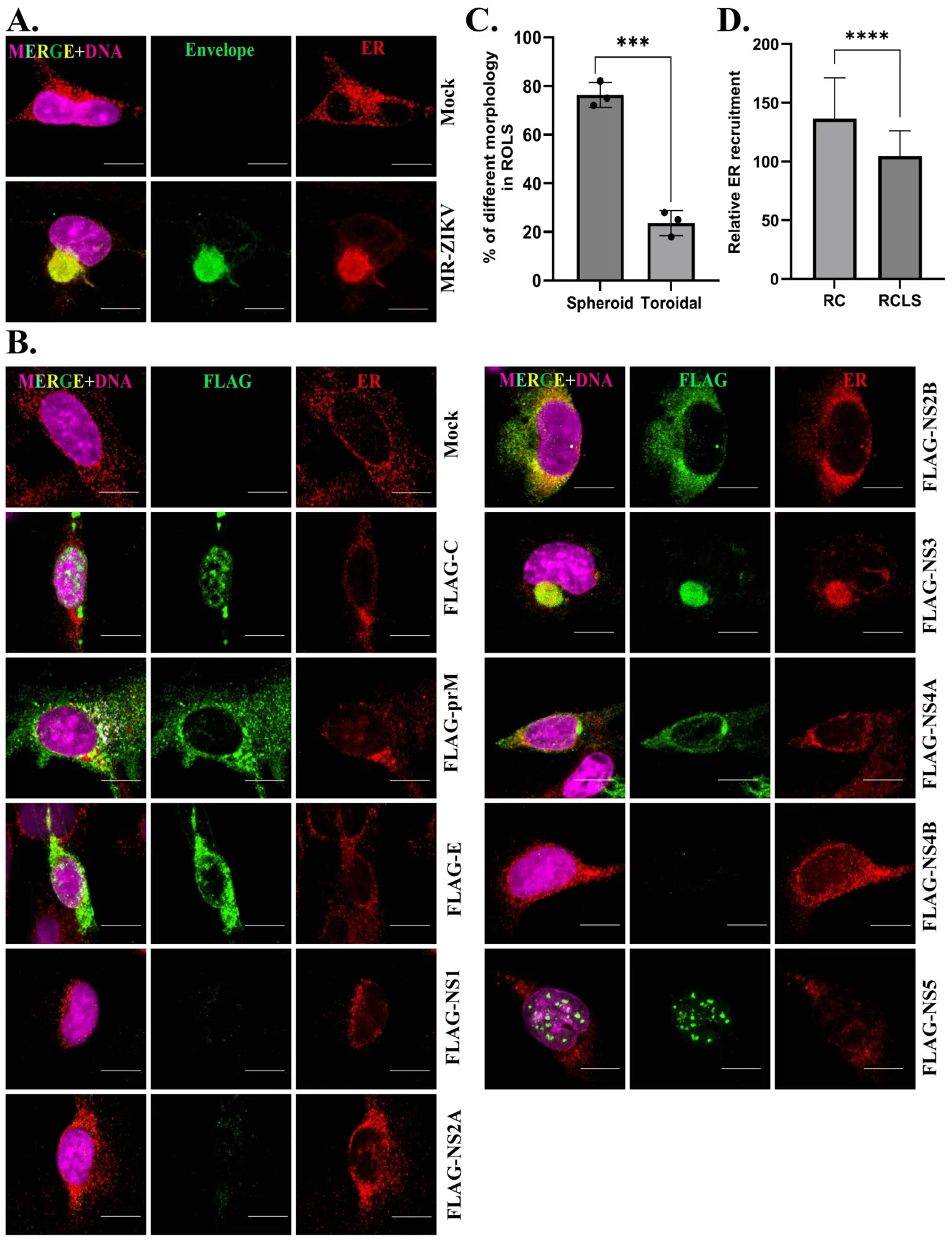
ZIKV NS3 drives assembly of a replication compartment-like structure (RCLS). Immunofluorescent (IF) staining of (**A**) mock (non-infected) and MR766 ZIKV (MR-ZIKV)-infected cells 24 h post-infection. RC is marked by ZIKV envelope protein (green) and ER is marked by calnexin (red). DNA is in magenta in all images. MOI = 1 was used for all infections. IF staining of (**B**) mock (non-transfected) and FLAG-tagged structural viral proteins: C (capsid), prM (pre-membrane), E (envelope), and non-structural (NS) protein expression in SNB-19 cells. NS1-, NS2A-, and NS4B-expressing plasmids did not yield detectable protein expression under these conditions. ER is marked by KDEL (red) and the NS3-driven RCLS is marked by FLAG (green). Scale bars: 10 μm. (**C**) Quantification of different morphology of the NS3-driven RCLSs. (**D**) Quantification of mean fluorescence intensity analysis of ER signal colocalizing with RCs and RCLSs. Representative images are based on *n* = 3 independent experiments; quantification was based on 25–30 images in total per condition. Unpaired Student’s *t*-test was carried out. *** *p* <  0.001, and **** *p* <  0.0001 are considered statistically significant.

The ER is typically a dispersed organelle in cells (Figure 1A,B). However, upon MR-ZIKV infection, ER reorganizes and is incorporated into the RC, a toroidal structure adjacent to the nucleus in human cells including SNB-19 cells, an astrocytoma cell line (Figure 1A), and HEK293T cells (Figure S1B). We transfected plasmids that express individual FLAG-tagged ZIKV proteins (C, prM, E, NS2B, NS3, NS4A, NS5) from MR-ZIKV in SNB-19 cells and found that NS3 was sufficient to generate a spherical compartment similar to the RC (Figure 1B). None of the other ZIKV proteins that we tested generated a structure resembling the RC when expressed from plasmids in cells (Figure 1B). We were unable to detect expressions of NS1, NS2A, and NS4B from the plasmids, so we cannot make conclusions about their involvement. We refer to the structure formed by NS3 as the replication compartment-like structure, or RCLS, because it recruits the ER and forms at the nuclear periphery, as does the virus-induced RC (Figure 1B).

ZIKV-induced RCs assemble around the centrosome and have a toroidal shape 96% of the time (Figure S1C) [22]. In contrast, the RCLS is toroidal 24% of the time and is spherical with no discernable hollow core at a frequency of 76% (Figure 1D and Figure S1D). Moreover, ER recruitment to the RC is robust compared to the NS3-driven RCLS (Figure 1D). This disparity suggests that ER recruitment during RC formation likely requires additional viral or host factors which are not available when NS3 alone assembles the RCLS.

### 3.2. The NS3-Driven RCLS Forms in Association with the Centrosome and Recruits Golgi

**Figure 2:**
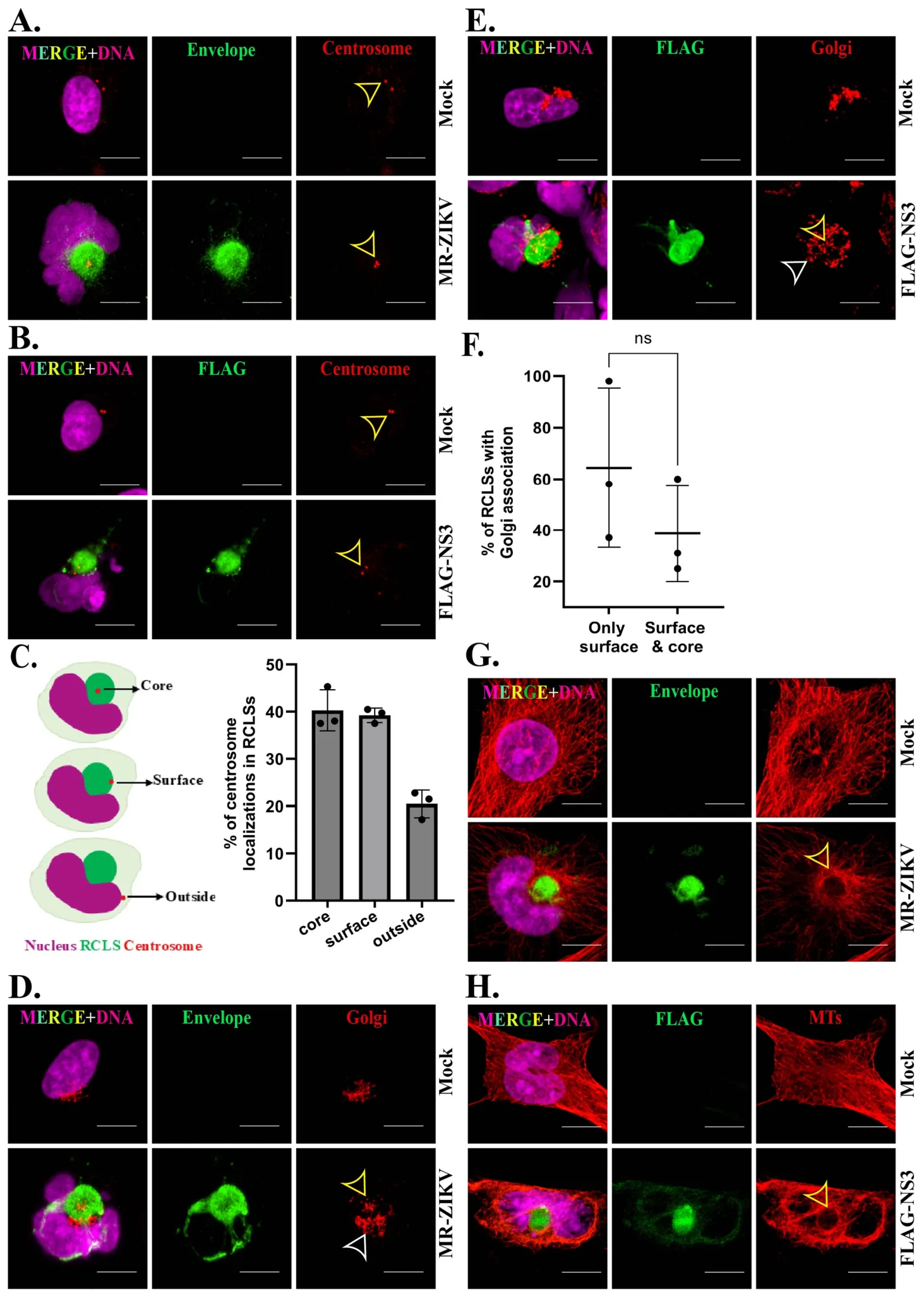
The NS3-driven RCLS forms in association with the centrosome and Golgi and reorganizes MTs. Immunofluorescent (IF) staining for the centrosome protein CEP192 (red) in (**A**) mock (non-infected) and MR766 ZIKV (MR-ZIKV) 24 h post-infection and (**B**) mock (non-transfected) and FLAG-NS3 transfected for 48 h in SNB-19 cells. The ZIKV-induced RC (green) and NS3-driven RCLS (green) are marked by envelope protein and FLAG, respectively, and DNA in magenta in all images. The localization of the centrosome is indicated by yellow arrowheads. (**C**) Illustration and bar graph (mean ±  standard deviation) shows different localization of the centrosome in NS3-driven RCLSs. Dots represent individual values from *n* = 3 independent experiments in all figures. IF staining of the Golgi protein GM130 (red) in (**D**) mock and MR-ZIKV infection for 24 h and (**E**) mock and FLAG-NS3 transfected for 48 h in SNB-19 cells. The Golgi at the core is indicated by yellow arrowheads and the Golgi surrounding the RC or NS3-driven RCLS by white arrowheads. (**F**) Quantification of Golgi pattern around the NS3-driven RCLS. IF staining of microtubules (MTs) marked by α-tubulin (red) in (**G**) mock and MR-ZIKV 24 h post-infection and (**H**) mock and FLAG-NS3 transfected for 48 h in SNB-19 cells. Yellow arrowheads indicate MTs around the RC and NS3-driven RCLS. Scale bars: 10 μm. MOI = 1 was used for all infections. Representative images are based on *n* = 3 independent experiments; quantification was based on 25–30 images in total per condition. Unpaired Student’s *t*-test was carried out. Not significant (ns).

The ZIKV RC forms around the centrosome, and Golgi associates with the RC surface [22]. We investigated whether these associations were also features of the NS3-driven RCLS and found that, similarly, the RCLS forms in conjunction with the centrosome, although the localization of the centrosome was not predominantly positioned in the center of the compartment as it typically is with the RC (Figure 2A–C). This difference in positioning of the centrosome likely accounts for the lower incidence of the toroidal over the spherical shape, as the centrosome is necessary for the hollow core of the toroid to form [22].

The Golgi apparatus is usually organized into a single compact organelle in SNB-19 cells (Figure 2D,E). In ZIKV-infected cells, the Golgi reorganizes into a more dispersed structure that is positioned on the surface and/or at the RC core (Figure 2D) [22]. We observed a similar reorganization of the Golgi and its localization at the surface of the NS3-driven RCLS and, less frequently, at the core (Figure 2E,F and Figure S1E).

### 3.3. Microtubules Are Reorganized at the NS3-Driven RCLS

The extensive MT array in mock-infected cells undergoes reorganization upon virus infection, adopting a cage-like formation surrounding the RC which was observed in 92% of infected cells (Figure 2G) [22], prompting us to explore the arrangement of MTs in the NS3-driven RCLS. We discovered that MTs also reorganized at the surface of the NS3-driven RCLS, encircling its surface similarly to the RC while forming a MT cage less frequently at 69 ± 17.5% (Figure 2H and Figure S1F).

### 3.4. Proper RCLS Formation by NS3 Requires Helicase Activity

**Figure 3:**
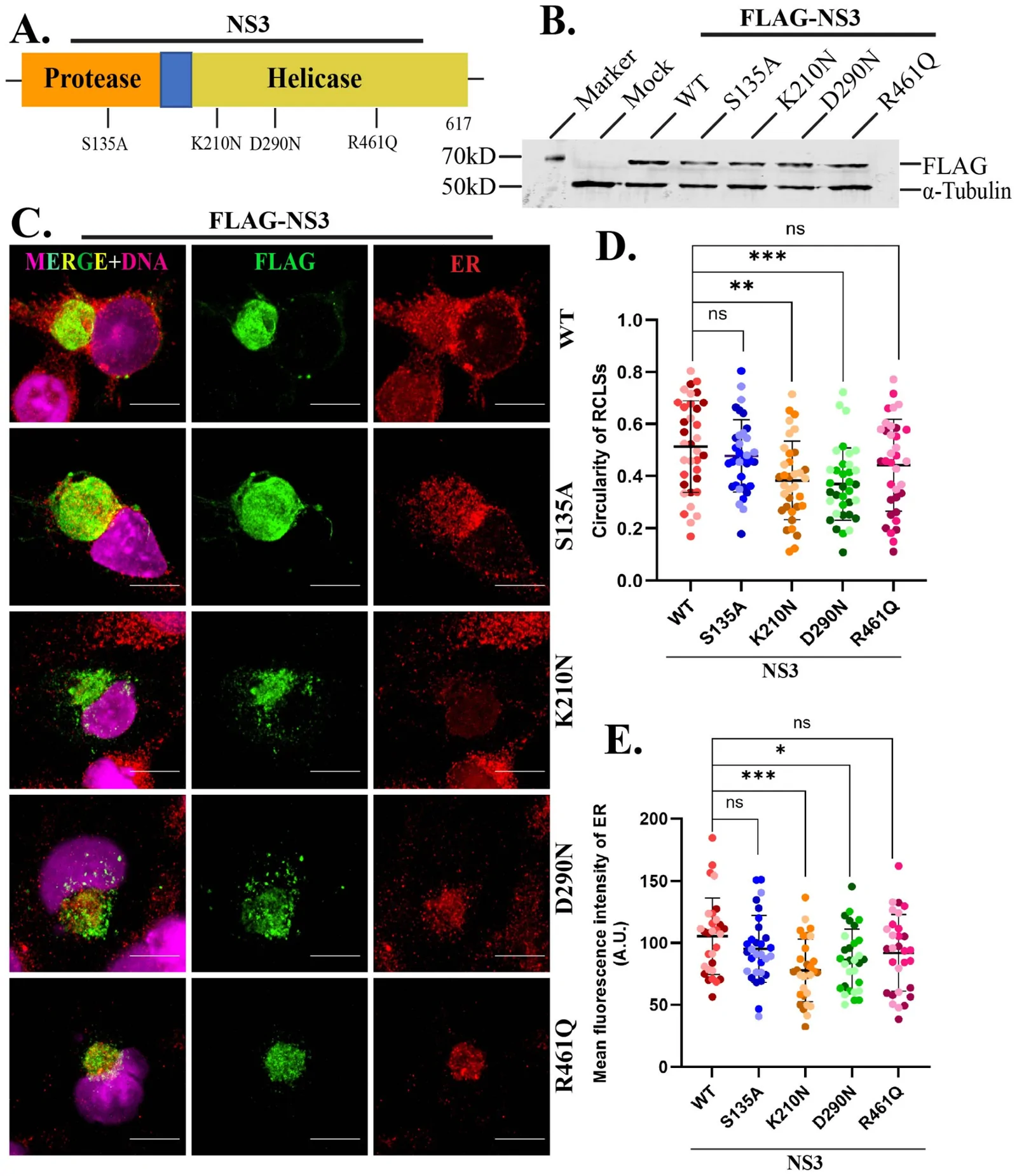
RCLS formation by NS3 requires the ATPase domain. (**A**) NS3 structure denoting protease and helicase domains and positions of introduced mutations. (**B**) Western blot of mock (non-transfected), FLAG-tagged wild-type (WT), and mutant NS3 (S135A, K210N, D290N, and R461Q) proteins in SNB-19 cells. (**C**) Immunofluorescent (IF) staining of WT and mutated NS3 transfected for 48 h in SNB-19 cells. NS3-driven RCLS is marked by FLAG (green) and ER is marked by KDEL (red). DNA is magenta. (**D**) Quantification of RCLS circularity with different NS3 mutations. Different colors indicate different experiments. Scale bars: 10 μm. (**E**) Quantification of mean fluorescence intensity analysis of ER signal colocalizing with NS3-driven RCLS transfected with WT and NS3 mutants. Representative images are based on *n* = 3 independent experiments; quantification was based on 25–30 images in total per condition. One-way ANOVA followed by Dunnett’s post hoc test for multiple comparisons was carried out. * *p* <  0.05, ** *p* <  0.01 and *** *p* <  0.001, are considered statistically significant. Not significant (ns).

Having demonstrated the sufficiency of NS3 to drive RCLS assembly, we next tested whether the protease or helicase activities of NS3 are required. To address this, we expressed the isolated NS3 protease and helicase domains (Figure S2A). The protease domain showed no detectable structures, whereas the helicase domain generated fragmented, discontinuous assemblies dispersed throughout the cytoplasm that did not generate a complete RCLS (Figure S2A), indicating that full-length NS3 is required for robust RCLS assembly. We next introduced mutations at functional residues of the protease or helicase domains of NS3 based on previous studies showing functional impairment from these mutations [36,37,38,39]. The mutations we introduced include a serine to alanine substitution (S135A) at the protease domain catalytic serine and, in the helicase domain, to impair ATP binding and hydrolysis, lysine to asparagine (K210N) in the Walker A motif, and aspartic acid to asparagine (D290N) in the Walker B motif, respectively, and changed an arginine essential for nucleic acid binding to glutamine (R461Q) in the helicase RNA-binding motif (Figure 3A). A summary of the mutants is listed in Table S2. All mutant proteins were expressed at similar levels to the wild-type NS3 protein in transfected cells (Figure 3B). We then investigated the impact of these mutations on RCLS formation.

All the mutants generated an RCLS to some degree, but the mutations in the ATPase Walker A and B motifs (K210N and D290N, respectively) produced less compact RCLSs (Figure 3C,D and Table S3). Mutations in the protease and RNA-binding domains (S135A and R461Q, respectively), on the other hand, resulted in no detectable morphological impairment in the ability of NS3 to assemble the RCLS (Figure 3C,D and Table S3).

### 3.5. NS3 Mutants Differentially Affect Organelle Recruitment to RCLSs

We assessed whether NS3 mutants affect organelle recruitment to the NS3-driven RCLS. The K210N and D290N mutants of the helicase domain showed markedly reduced ER recruitment compared to wild-type NS3, indicating that the ATPase activity of NS3 is a key determinant for efficient ER recruitment to RCLSs (Figure 3C,E and Table S3). In contrast, the S135A and R461Q mutants did not significantly impair ER association with the RCLS. This effect could be related to, or a consequence of, the less-compact RCLS formed by the K210N and D290N mutants (Figure 3C,D).

**Figure 4:**
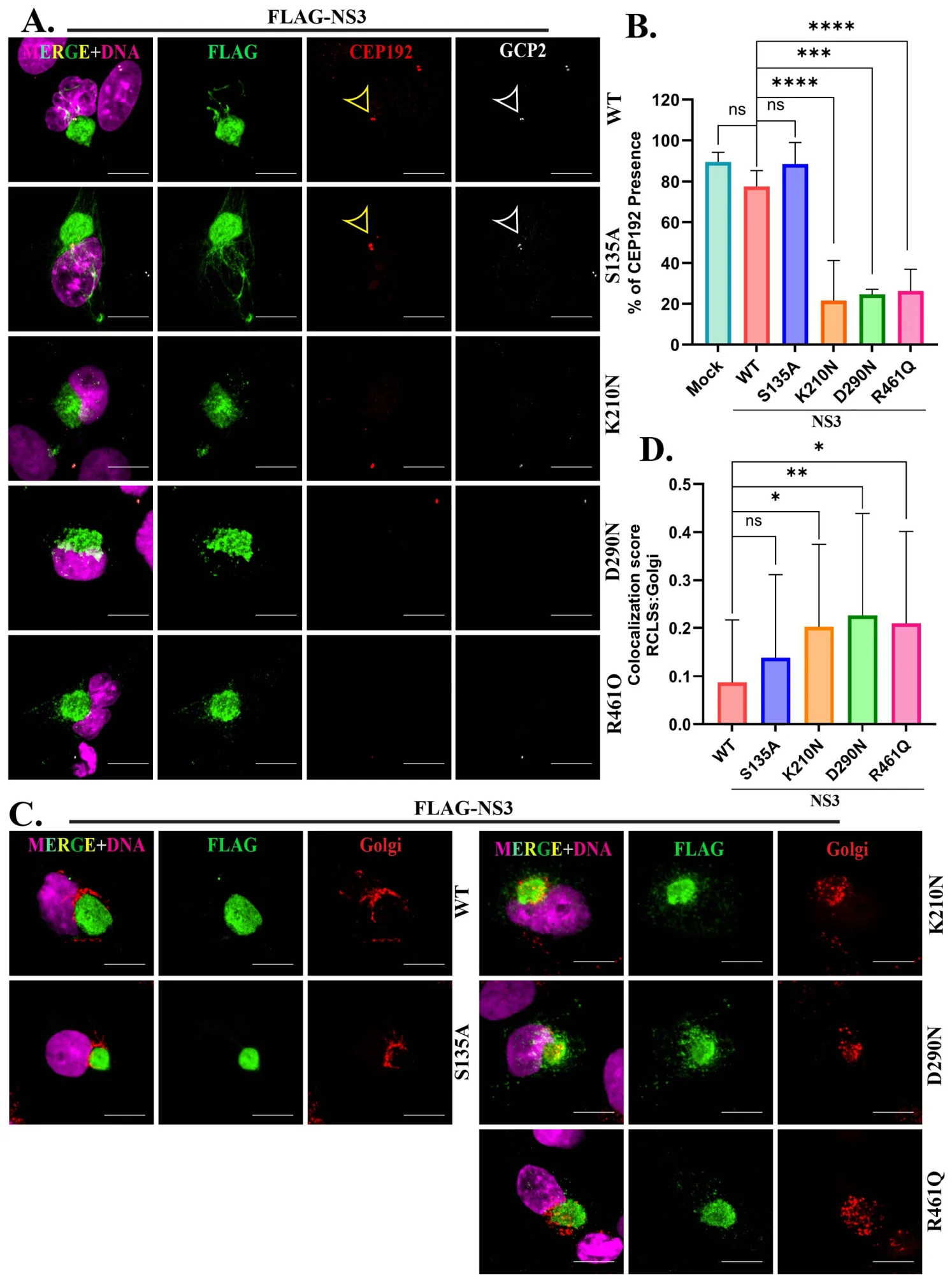
Impact of NS3 mutants on centrosome and Golgi recruitment. (**A**) Immunofluorescent (IF) staining of cells expressing wild-type (WT) and mutant NS3 (S135A, K210N, D290N, and R461Q) transfected for 48 h in SNB-19 cells with centrosomal protein CEP192 (red) and GCP2 (white). NS3-driven RCLS is marked by FLAG (green) and DNA in magenta in all images. Yellow and white arrowheads indicate CEP192 and GCP2 localization, respectively, in NS3-driven RCLSs. (**B**) Quantification of CEP192 localization in NS3-driven RCLSs formed by mock (non-transfected), WT, and mutant NS3. (**C**) IF staining of WT and mutated NS3 transfected for 48 h in SNB-19 cells with Golgi protein GM130 (red). (**D**) Quantitative analysis of GM130 colocalization with RCLSs in cells expressing WT or mutant NS3. Scale bars: 10 μm. Representative images are based on *n* = 3 independent experiments; quantification was based on 25–30 images in total per condition. One-way ANOVA followed by Dunnett’s post hoc test for multiple comparisons was carried out. * *p* <  0.05, ** *p* <  0.01, *** *p* <  0.001, and **** *p* <  0.0001 are considered statistically significant. Not significant (ns).

We investigated the impact of NS3 mutants on the association of the centrosome with the RCLS. We found that localized signals for CEP192 and GCP2, two core components of the centrosome, were significantly absent in cells expressing the K210N, D290N, and R461Q mutants (Figure 4A,B and Figure S2B and Table S3) compared to wild-type NS3 and the S135A mutant. These findings suggest that the ATPase and RNA-binding NS3 mutants interfere with the recruitment of centrosomal proteins to centrosomes or disrupt centrosome assembly or maintenance. This was unexpected, as we did not anticipate the composition or integrity of the centrosome to be impacted by the expression of NS3 mutants given that expression of wild-type NS3 had no apparent effect.

We next evaluated Golgi apparatus localization in cells expressing NS3 mutants. In cells expressing wild-type NS3 and the S135A mutant, the Golgi was primarily arranged around the periphery of the RCLS (Figure 4C and Table S3). In contrast, cells expressing either of the three NS3 helicase mutants exhibited enhanced colocalization of the Golgi within the RCLS interior rather than localizing to the RCLS surface (Figure 4C,D and Table S3). It therefore appears that NS3 helicase mutant RCLSs’ can recruit Golgi, but do not organize it on the surface like wild-type NS3 (or the S135A mutant).

**Figure 5:**
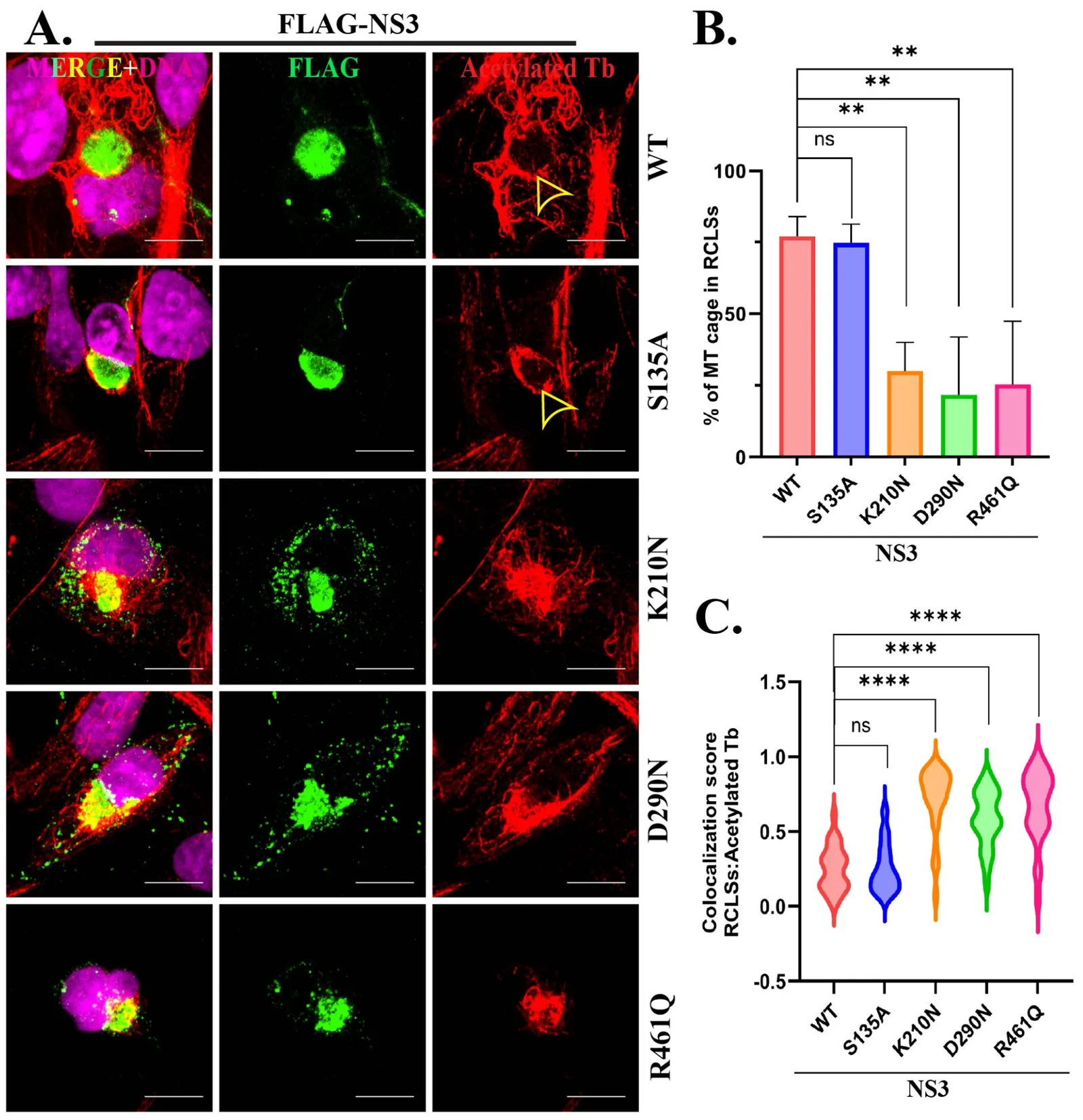
Impact of NS3 mutants on microtubule organization. (**A**) Immunofluorescent (IF) staining of wild-type (WT) and mutated NS3 transfected for 48 h in SNB-19 cells marked by acetylated tubulin (Tb) (red). NS3-driven RCLS is marked by FLAG (green) and DNA is magenta. Yellow arrowheads indicate microtubules (MTs) in the cage-like structure around the NS3-driven RCLSs. (**B**) Quantification of MT cage-like structure in NS3-driven RCLSs formed by WT and mutant NS3. (**C**) Quantification of acetylated MT colocalization with RCLSs formed by NS3 WT and mutants. Scale bars: 10 μm. Representative images are based on *n* = 3 independent experiments; quantification was based on 25–30 images in total per condition. One-way ANOVA followed by Dunnett’s post hoc test for multiple comparisons was carried out. ** *p* <  0.01, and **** *p* <  0.0001 are considered statistically significant. Not significant (ns).

We examined MT organization in cells transfected with NS3 mutants. Unlike the well-defined cage-like MT arrangements observed in cells expressing wild-type NS3 and the S135A mutant, the K210N and R461Q mutants exhibited a significant reduction in MT organization surrounding the RCLS. The D290N mutant also displayed a downward trend in organization; however, this decrease did not reach statistical significance (Figure 5A,B and Table S3). Additionally, acetylated MTs were significantly enriched within the RCLSs formed by the NS3 helicase mutants (Figure 5A,C and Table S3). These findings suggest that the NS3 helicase domain prevents the accumulation of stable MTs within the RCLS, ensuring a dynamic and organized MT architecture surrounding the RCLS.

### 3.6. ZIKV NS3 Activates the UPR but Also Blocks CHOP, Requiring NS3 Protease and Helicase Activities

**Figure 6:**
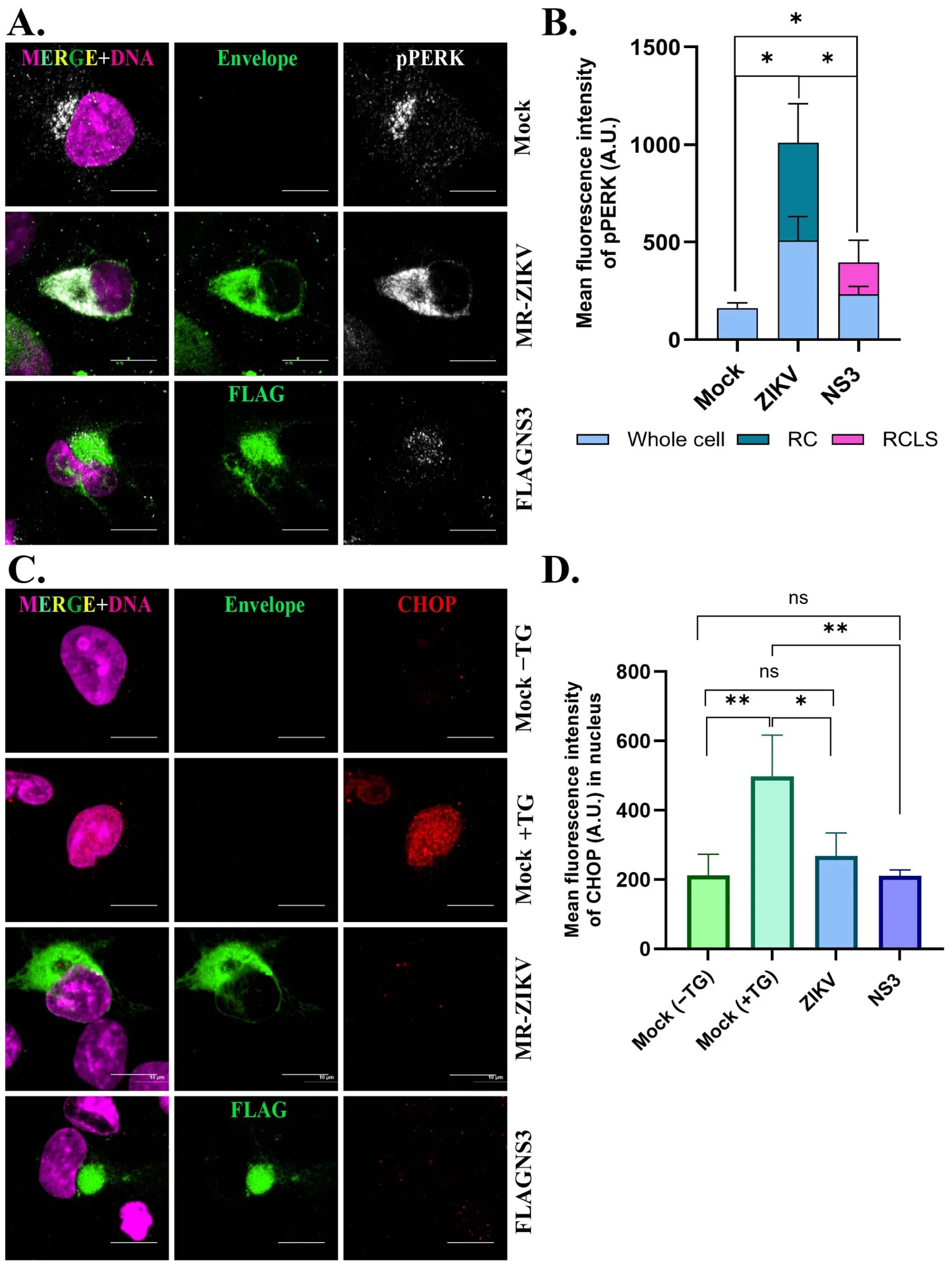
ZIKV and NS3 block CHOP. (**A**) Immunofluorescent (IF) staining for pPERK (white) in (**A**) mock (non-infected) and MR766 ZIKV (MR-ZIKV) 24 h post-infection and FLAG-NS3 transfected for 48 h in SNB-19 cells. ZIKV-induced RC (green) and NS3-driven RCLS (green) are marked by envelope protein and FLAG, respectively, And DNA in magenta in all images. (**B**) Quantification of mean fluorescence intensity of pPERK signal. (**C**) IF staining for CHOP (red) in mock (non-infected), MR-ZIKV 24 h post-infection, and FLAG-NS3 (green) transfected for 48 h in SNB-19 cells. Indicated cells were treated with 1µM of thapsigargin (TG) for 4 h. (**D**) Quantification of mean fluorescence intensity of CHOP signal in SNB19 cells. Scale bars: 10 μm. Representative images are based on *n* = 3 independent experiments; quantification was based on 25–30 images in total per condition. Unpaired Student’s *t*-test was carried out. * *p* <  0.05 and ** *p* <  0.01, are considered statistically significant. Not significant (ns).

Prior studies showed that ZIKV infection activated the UPR through PERK and other pathways [20], but inhibited expression of CHOP post-transcriptionally [19]. We confirmed this, showing that phospho-PERK (pPERK) is elevated in ZIKV-infected cells, and also in NS3-expressing cells (Figure 6A,B). In addition, pPERK is predominantly localized at the RC or RCLS (Figure 6A,B, Table S3). In control cells, thapsigargin treatment activated the UPR, resulting in elevated CHOP expression in the nucleus (Figure 6C,D), but did not impose overt change in the ER morphology at this resolution, indicating that ER stress was not sufficient to generate an RCLS (Figure S2C). Similar to ZIKV infection, expression of NS3 resulted in blocked CHOP expression (Figure 6C,D, Table S3). Therefore, NS3 is not only sufficient to construct an RCLS, but it also elicits the activation of the UPR with the selective repression of CHOP seen with ZIKV infection.

**Figure 7:**
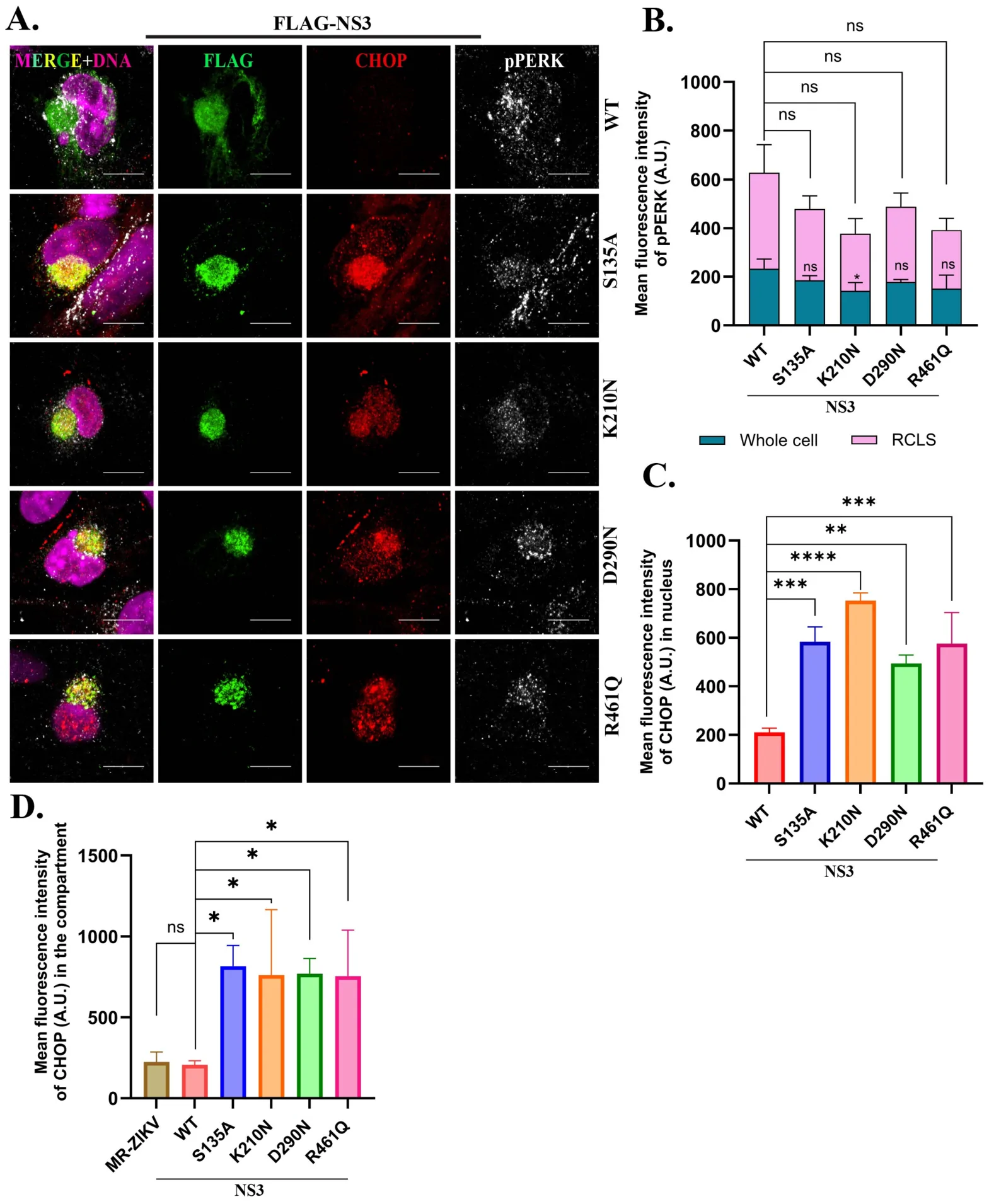
NS3 mutants lose the ability to suppress CHOP. (**A**) Immunofluorescent (IF) staining of SNB-19 cells transfected to express FLAG-tagged wild-type (WT) NS3 and mutants (S135A, K210N, D290N, and R461Q) to detect CHOP (red) and pPERK (white). NS3-driven RCLS is marked by FLAG (green). DNA is magenta. Quantification of mean fluorescence intensity of (**B**) pPERK, (**C**) CHOP in nucleus, and (**D**) CHOP in RCLS in SNB19 cells. Scale bars: 10 μm. Representative images are based on *n* = 3 independent experiments; quantification was based on 25–30 images in total per condition. One-way ANOVA followed by Dunnett’s post hoc test for multiple comparisons was carried out. * *p* <  0.05, ** *p* <  0.01, *** *p* <  0.001, and **** *p* <  0.0001 are considered statistically significant. Not significant (ns).

We further tested the contributions of the protease and helicase functions of NS3 to the UPR and found involvement of both domains. All four of the mutants induced the UPR similarly to wild-type NS3, with elevated pPERK in the RCLS, but all four mutants were deficient in inhibiting CHOP expression (Figure 7A, Table S3). While the NS3 S135A protease mutant showed no effect on RCLS formation and all other attributes assessed above, it was unable to block the expression of CHOP in the nucleus (Figure 7A,C, Table S3). Notably, all four NS3 mutants not only permitted CHOP expression, but partially retained it at the RCLS (Figure 7A,D, Table S3). This indicates that the protease and helicase mutants have a neomorphic role in the retention of CHOP at the RCLS, an aspect not seen with wild-type NS3.

### 3.7. NS3 Assembles the RCLS Independent of Its Partner NS2B

**Figure 8:**
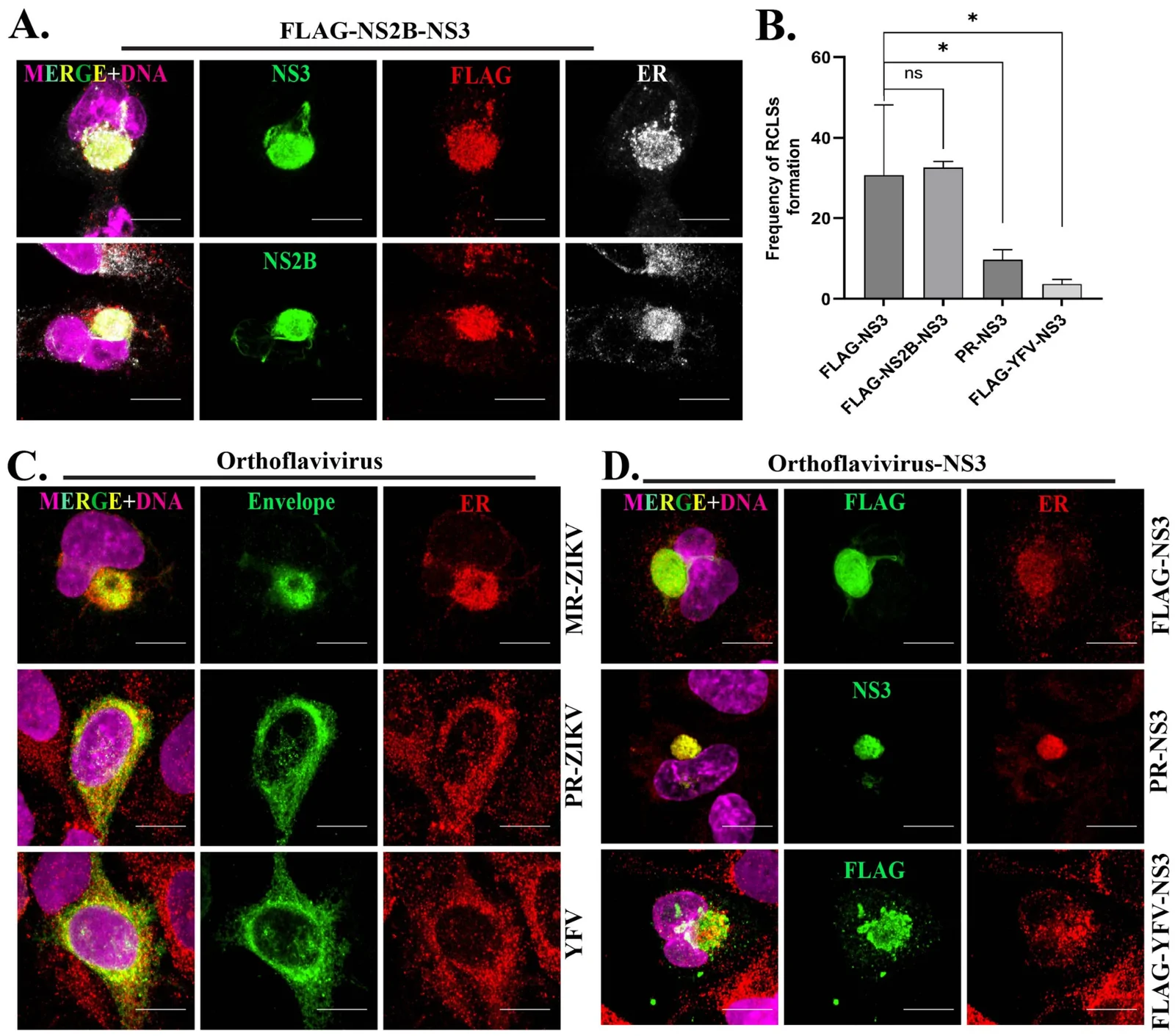
Other orthoflavivirus NS3 proteins can also form an RCLS. (**A**) Immunofluorescent (IF) staining of FLAG-NS3 and FLAG-NS2B–NS3 transfection in SNB-19 cells for 24 h. RCLS is marked by NS2B or NS3 (green) and FLAG (red). ER is marked by KDEL (white) and DNA is magenta in all images. (**B**) Quantification of the frequency of RCLS formation at 24 h by FLAG-NS3 and the fusion FLAG-NS2B–NS3, PRVABC59 ZIKV-NS3 (PR-NS3), and FLAG-Yellow fever virus (YFV)-17D-NS3 (FLAG-YFV-NS3). (**C**) IF staining of MR766 ZIKV (MR-ZIKV), PRVABC59 ZIKV (PR-ZIKV), and YFV-17D-204 (YFV) infected for 24 h in SNB-19 cells. Envelope is green and ER is marked by calnexin (red). MOI = 1 was used for all infections. (**D**) IF staining of FLAG-NS3, PR-NS3, and FLAG-YFV-NS3 transfection for 48 h in SNB-19 cells. NS3-driven RCLS is marked by FLAG or NS3 (green). ER is marked by KDEL (red), Scale bars: 10 μm. Data and representative images are based on *n* = 3 independent experiments. Unpaired Students’ *t*-test was carried out. * *p* < 0.05 are considered statistically significant. Not significant (ns).

NS2B is a direct partner of NS3 and functions as a protease cofactor with NS3 during viral replication [40]. To examine whether NS2B influences RCLS formation, we generated a FLAG-tagged NS2B–NS3 fusion construct. Western blot analysis detected three bands corresponding to NS2B, NS3, and the uncleaved NS2B–NS3 fusion protein, indicating partial self-cleavage of the fusion into its individual components (Figure S2D). Upon transfection into SNB19 cells, the NS2B–NS3 fusion protein efficiently induced RCLS formation (Figure 8A). However, NS2B alone was unable to drive RCLS formation, whereas NS3 was sufficient (Figure S2E). Notably, the frequency of RCLS formation in cells expressing NS2B–NS3 was comparable to that observed with FLAG-NS3 alone (Figure 8B), indicating that the presence of NS2B does not enhance NS3-mediated RCLS formation.

### 3.8. The MR-ZIKV Strain Is Unique in Forming the Toroidal RC, but Other Orthoflavivirus NS3 Proteins Can Also Form an RCLS

We compared SNB-19 cells infected with MR-ZIKV with another ZIKV strain, PRVABC59 (PR-ZIKV), and with yellow fever virus, 17D-204 (YFV), a different orthoflavivirus (Figure 8C). PR-ZIKV is from an Asian strain isolated during the 2015–2016 outbreak in the Americas and MR766 is of African origin, isolated in 1947. The YFV strain is an attenuated virus used for vaccination and has one amino acid change in the helicase domain [41,42,43].

Among these three orthoflaviviruses, only the MR-ZIKV consistently formed a discrete toroidal RC structure, indicating differences among ZIKV strains and orthoflavivirus species in RC structure and shape. To assess whether expression of NS3 from these viruses is sufficient to drive RCLS formation, we transfected cells with PR-NS3 and YFV-NS3. In both cases, NS3 expression led to the formation of a spherical RCLS, albeit at a lower frequency, and recruited ER components (Figure 8B,D and Figure S2D,F), indicating that NS3-driven RCLS formation and ER recruitment may be a conserved feature among orthoflavivirus NS3 proteins.

## 4. Discussion

ZIKV forms ER-membrane invaginations similar to those generated by DENV [16], but can also induce more extensive reorganization of the ER when it organizes the RC. Here we show that the ZIKV NS3 is sufficient to assemble a compartment within human cells with all the features of an RC, which we refer to here as the RCLS.

During MR-ZIKV infection, the mature RC typically exhibits a toroidal morphology, and a spherical shape is found less frequently. In contrast, the NS3-driven RCLS more often has a spherical shape (Figure 1C and Figure S1C,D). While NS3 expression in the absence of other ZIKV-encoded proteins can initiate RCLS formation, it likely lacks additional viral and host machinery to fully establish the architecture of the mature RC. Previous investigations have also shown colocalization of orthoflavivirus NS3 with the ER [44,45,46]. However, in contrast with the dynamics of RC formation during ZIKV infection, the RCLS recruits less ER (Figure 1D), indicating that ER recruitment may not be limiting for RCLS assembly. One possible explanation is that during viral replication, native NS3 is expressed proximally to the ER as the ZIKV genome is translated as a polyprotein at the ER and processed there during RC formation. In contrast, the plasmid-expressed FLAG-NS3 protein deployed in this study is not co-translationally targeted to the ER and does not acutely localize there. This could partially explain the lower ER recruitment observed at the RCLS relative to the RC. While these data indicate that NS3 is sufficient to generate a RCLS and recruit ER, other ZIKV proteins might be needed for full efficiency and processing at the ER, but NS2B, an established NS3 partner, does not appear to augment this activity. One limitation of our study is that we were unable to detect NS1, NS2A and NS4B expression from plasmids.

The morphology of RCs differed between MR-ZIKV, PR-ZIKV, and YFV infections, yet expression of NS3 from PR-ZIKV or YFV still led to RCLS formation with ER recruitment. ZIKV strains MR766 and PRVABC59 belong to distinct lineages (African vs. Asian, respectively) and differ at multiple amino acid positions across structural and non-structural proteins [47,48], which therefore likely contribute to the strain-dependent RC phenotypes observed here. The YFV strain used here (YFV-17D-204) is an attenuated virus for vaccine development and has low virulence. It has one mutation in NS3 (D485N) which might contribute to its reduced virulence [41,42,43], and possibly also reduce the capacity of the virus to form a spherical or toroidal RC, but that idea remains untested. Nonetheless, the core functions of NS3 are conserved across orthoflaviviruses [26,49,50], suggesting why PR-ZIKV-NS3 and YFV-NS3 can induce RCLS formation with ER recruitment despite lineage divergences.

ZIKV protease activity relies upon the NS2B–NS3 partnership; however, RCLSs formed by NS3 and the NS2B–NS3 fusion occurred at a similar frequency, indicating that NS2B is not required for, nor does it enhance, NS3-driven RCLS assembly. NS2B is an established cofactor for the NS3 protease, but in at least one case NS3 protease activity toward host proteins has been demonstrated to occur in the absence of NS2B [51]. The NS3 S135A mutant in the protease domain did not impact RCLS formation or most aspects of the RCLS that we measured, but it was unable to block CHOP nuclear localization, suggesting a specific role for the protease in suppressing CHOP.

In ZIKV-infected cells, the centrosome typically lies at the core of the RC and is associated with acetylated MTs [22] and Golgi. However, the NS3-driven RCLS lacks the characteristic central location of the centrosome (Figure 2A–C) and Golgi (Figure 2D,F and Figure S1E). We previously showed that elimination of the centrosome from cells did not impede RC formation but generated a spheroid rather than toroidal RC [22]. Since NS3 overexpression results in mostly spheroid RCLSs, this is likely a result of centrosome inactivation, a conclusion consistent with prior findings that NS3 associates with CEP63 and disrupts the centrosome [52].

Many different viruses rely on and alter MTs to facilitate the intracellular trafficking of virus components [53,54,55,56,57,58,59,60,61], including orthoflaviviruses [16,22,62,63,64,65]. MT arrays were shown to be localized at the mature RCs of various orthoflaviviruses such as DENV, Kunjin virus, ZIKV, and tick-borne encephalitis virus [16,22,66,67,68], and NS3 of JEV and ZIKV were found to be associated with MTs [16,44,69,70]. It was predicted that NS3 might induce MT reorganization in JEV-infected cells in order to facilitate the intracellular transport of other viral proteins during JEV replication [44]. Our previous findings along with other investigations reveal that MTs form a cage-like arrangement in close proximity to the mature RC in ZIKV-infected cells [16,22]. As with the RC upon ZIKV infection, MTs surrounded the RCLS in a cage-like structure (Figure 2H and Figure S1F). The organization of MTs at the surface of the RCLS indicates that NS3 is sufficient for the recruitment of factors that control MT nucleation, stability, or anchoring at the RCLS.

To further understand the mechanisms by which NS3 can organize the RCLS compartment, we expressed NS3 protease and helicase domains separately, and NS3 with mutations that impair the protease, helicase ATPase, and RNA binding activities [36,37]. Neither isolated domain alone was sufficient to generate an RCLS, indicating that full-length NS3 is required for compartment formation. As described above, the S135A mutation in the protease catalytic site did not affect RCLS formation, except for impairing the ability of NS3 to block CHOP expression. Mutations that disrupted the helicase domain affected the morphology of the RCLS, but did not disrupt its formation, yet Golgi recruitment and MT organization at its surface were altered, and instead were focused at the interior of the RCLS. While wild-type NS3 mimicked the ability of ZIKV infection to activate the UPR while blocking downstream CHOP expression, all four of the mutants retained UPR activation, but they no longer blocked CHOP expression. Remarkably, the helicase domain mutants displayed neomorphic features, including the blockage of centrosome assembly, elevated MT organization at the core of the RCLS, and the localization of CHOP at the RCLS (the protease mutant also shared this feature). Therefore, the helicase domain is uniquely involved in the assembly and function of the RCLS, including MT organization and CHOP dynamics. How these NS3 mutants affect the centrosome is more enigmatic. Additional studies will be necessary to understand the mechanisms and host factors that contribute to these functions of NS3 to control the virus replication compartment and manipulate host functions.

## Data Availability

The data presented in this study is available upon request from the corresponding author.

## Supplementary Materials

The following supporting information can be downloaded at: https://www.mdpi.com/article/10.3390/v18080834/s1, Figure S1: Morphology of RC and RCLS; Figure S2: Sufficiency of NS3 domain for RCLS formation and self-cleavage of NS2B–NS3. Table S1: Primers for cloning and mutagenesis of NS3. Table S2: Summary of NS3 mutants. Table S3: Summary of effects of ZIKV NS3 mutants on RCLS formation and organelle organization.

## Abbreviations

The following abbreviations are used in this manuscript:

C	Capsid
DENV	Dengue virus
ER	Endoplasmic reticulum
E	Envelope
FBS	Fetal bovine serum
H	Hour
JEV	Japanese encephalitis virus
MOI	Multiplicity of infection
NS	Non-structural
PBS	Phosphate-buffered saline
prM	Pre-membrane
ROIs	Regions of interests
RC	Replication compartment
RCLS	Replication compartment-like structure
RT	Room temperature
TBST	Tris-Buffered Saline with Tween-20
YFV	Yellow fever virus
ZIKV	Zika virus
