# Supplementary material for: Zika Virus NS3 Drives the Assembly of a Replication Compartment-like Structure That Exerts the Structural and Physiological Functions of the Viral Replication Compartment
Source: Viruses. 2026 Jul 29;18(8):834. doi: 10.3390/v18080834 (PMC13517270; doi:10.3390/v18080834)
Supplement: Supplementary file 1 [file viruses-18-00834-s001.zip › viruses-4311933-supplementary.pdf]

viruses

MDPI

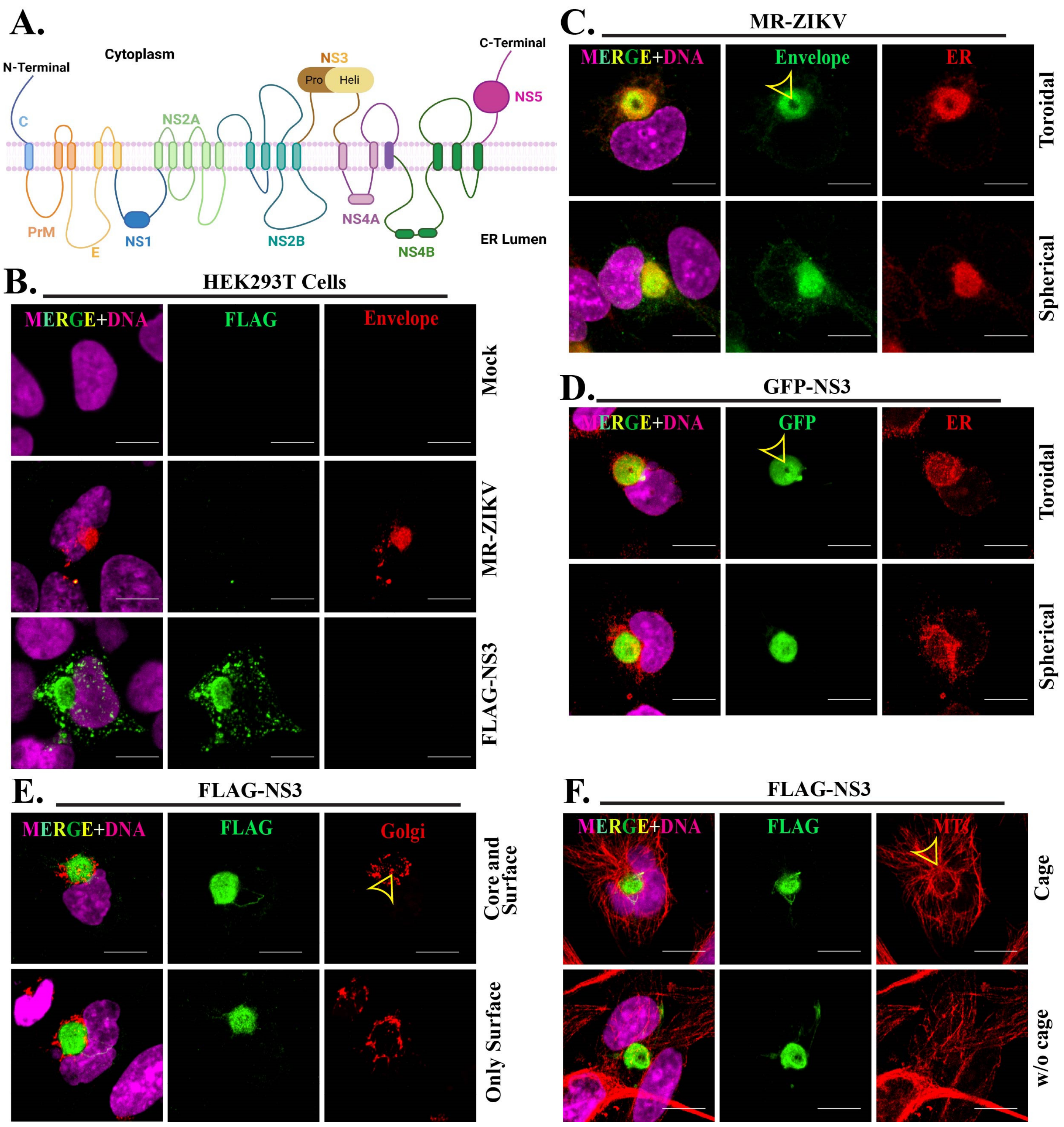

Figure S1. Morphology of RC and RCLS.

(A) Schematic image of the ZIKV polyprotein and its topology within the ER membrane. Created with Biorender.com. Agreement number: PG28EH5H14. Immunofluorescent (IF) staining of (B) mock (upper panel) and ZIKV-infected (middle panel) for 24h post-infection (RC marked by ZIKV envelope protein; red and FLAG-NS3 transfected (bottom panel) for 48h (RCLS marked by FLAG; green) in HEK293T cells. Morphology of the (C) RC marked by Envelope (green) and (D) NS3-driven RCLSs marked by GFP (green). The core of the RCLS when it takes the toroidal shape is marked by

 https://doi.org/10.3390/xxxxx

a yellow arrowhead. (E) Arrangement of Golgi protein GM130 (red) in the NS3-driven RCLS (; green,) transfected for 48h in SNB-19 cells. The Golgi at the core is indicated by yellow arrowheads. (F) IF staining of the microtubule (MT) arrangements marked by anti-tubulin (red) in NS3-driven RCLS (FLAG; green) transfected for 48h in SNB-19 cells. The MT cage structure is indicated by yellow arrowheads. Scale bars: 10 μm. Representative images are based on n = 3 independent experiments.

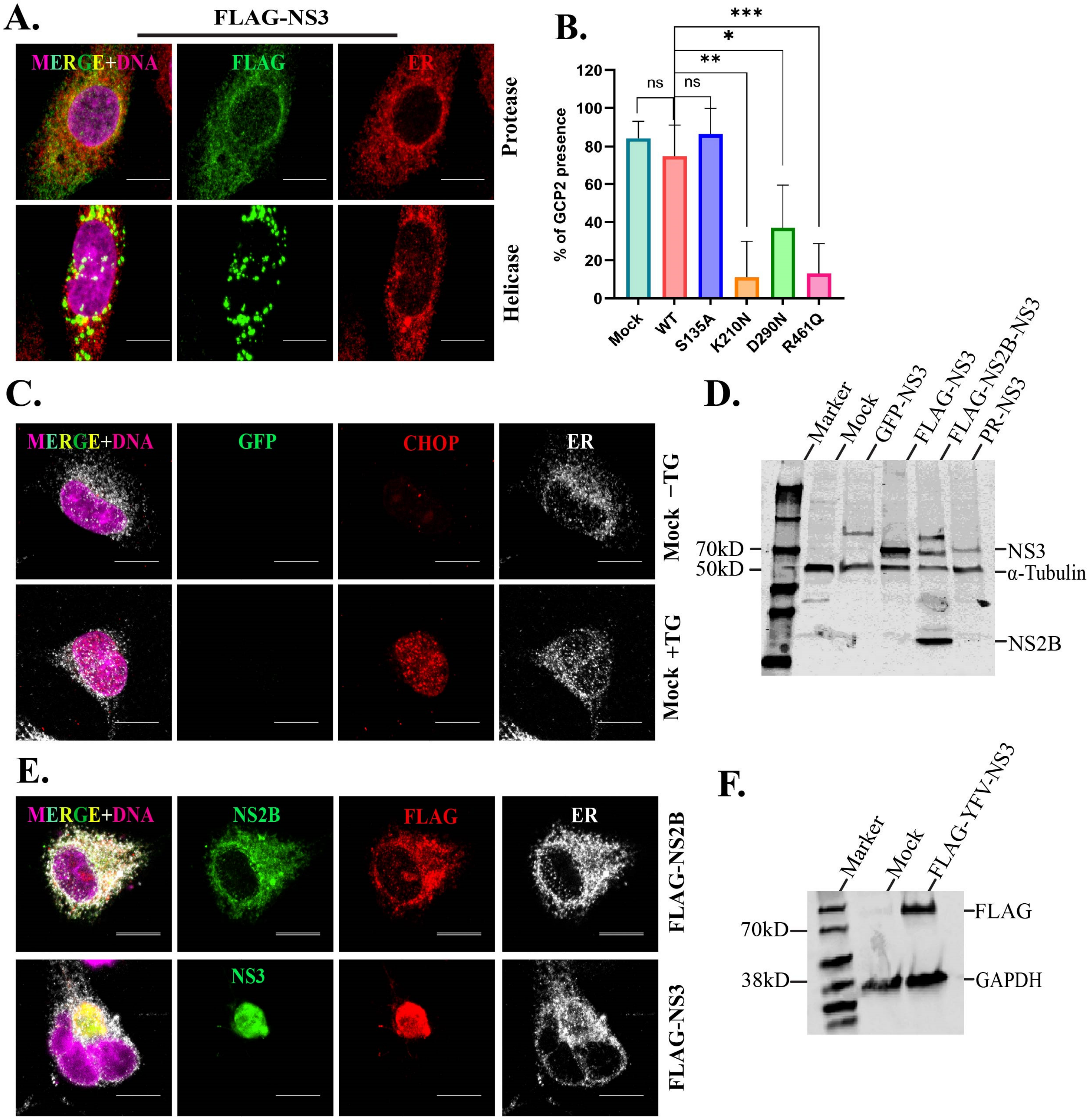

Figure S2. Sufficiency of NS3 domain for RCLS formation and self-cleavage of NS2B–NS3.

(A) Immunofluorescent (IF) staining of the individual transfection of NS3-protease and helicase domain in SNB19 cells. FLAG is green and ER is marked by KDEL. (B) Quantification of GCP2 presence in RCLSs formed by mock (not-transfected), NS3 Wild-type (WT) and mutants. (C) IF staining of

thapsigargin (+/-TG) treated mock (not-transfected) SNB-19 cells (GFP; green). Indicated cells were treated with 1μM of thapsigargin for 4hr. Unfolded protein response is marked by CHOP (red) and ER is marked by calnexin). (D) Western blot of various NS3 constructs in HeLa cells. IF staining of (E) FLAG-NS2B and FLAG-NS3 transfection in SNB-19 cells for 24h. RCLS is marked by NS2B or NS3 (green) and FLAG (red). ER is marked by KDEL (white). (F) Western blot of YFV-NS3 expression in HeLa cells. Mock cells were used as control in all Western blots. Scale bars: 10 μm. Data and representative images are based on n = 3 independent experiments. One-way ANOVA followed by Dunnett's post-hoc test for multiple comparisons was carried out. $^{*}P < 0.05$, $^{**}P < 0.01$, $^{***}P < 0.001$ and $^{****}P < 0.0001$ are considered statistically significant. Not significant (ns).

Table S1. Primers for cloning and mutagenesis of NS3

Primer name	Sequence (5′-3′)
ZIKV-NS3-F	CACCAGTGGTGCCCTCTGGGA
ZIKV-NS3-R	TTATCTTTTTCCAGCGGCGAA
ZIKV-NS3-Protease-F	CACCAGTGGTGCCCTCTGGGA
ZIKV-NS3-Protease-R	CTACTTCAGCATCGAGGGTTC
ZIKV-NS3-Helicase-F	CACCGGAAAGAGGGAGGAGGAGAC
ZIKV-NS3-Helicase-R	TTATCTTTTTCCAGCGGCGAA
NS3_S135A-F	TACCCTGCAGGGACCGCAGGATCTCCGATCC
NS3_S135A-R	GGATCGGAGATCCTGCGGTCCCTGCAGGGTA
NS3_K210N-F	TCCAGGAGCCGGAAATACCAGGAGAGTTCTT
NS3_K210N-R	AAGAACTCTCCTGGTATTTCCGGCTCCTGGA
NS3_D290N-F	AATCTCTACATCATGAATGAAGCCCACTTCA
NS3_D290N-R	TGAAGTGGGCTTCATTCATGATGTAGAGATT
NS3_R461Q-F	GCTAGTGCTGCTCAGCAGAGAGGACGTATAGG
NS3_R461Q-R	CCTATACGTCCTCTCTGCTGAGCAGCACTAGC
NS2B-NS3-F	CACCATGAGCTGGCCCCCTAGTGA
NS2B-NS3-R	TAATCTTTTTCCAGCGGCGAATTCT
YFV-NS3-F	CACCATGAGTGGGGATGTCTTGTGGGAT
YFV-NS3-R	TAACCTCCTACCTTCAGCAAACTT

Non-structural (NS); Yellow fever virus (YFV); Forward (F); Reverse (R)

Table S2. Summary of NS3 mutants

Mutated amino acids (aa)	Domain	Motif	Consensus sequence (mutated aa in bold)	Function	References
S135A	Protease	Chymotrypsin-like catalytic serine	Gx[DE]x**S**Gx[GS]	Cleaves the viral polyprotein	[36,38]

K210N	Helicase	Walker A	AxxGxG**K**S/T	ATP binding	[37,39]
D290N		Walker B	**D**ExH	ATP hydrolysis	
R461Q		Motif VI	Q**R**xGRxGR	Nucleic acid binding	

Table S3. Summary of effects of ZIKV NS3 mutants on RCLS formation and organelle organization

Condition / Construct	RCLS Formation	RCLS Morphology	ER Recruitment	Centrosome Association (CEP192/GCP2)	Golgi Localization	MT Organization	UPR Activation
Mock / No transfection	None	Not applicable	Normally dispersed ER	Normal centrosome	Compact Golgi	Normal MT network	Normal pPERK and no CHOP expression. Increased CHOP in nucleus with the treatment of TG
MR-ZIKV infection	RC	toroidal > spherical	Strong	Centrosome at RC core	Surface + core	Strong MT cage	High pPERK in RCLS but blocks CHOP
WT NS3	RCLS	spherical > toroidal	Moderate	Varied localization (often off-center)	Mainly surface	MT cage	Moderate pPERK in RCLS but blocks CHOP
S135A (Protease mutant)	RCLS	Similar to WT	Similar to WT	Similar to WT	Similar to WT	Similar to WT	pPERK: similar to WT<br>CHOP: significantly high in nucleus and RCLS
K210N (Walker A – ATP binding)	RCLS	Significantly less compact	Significantly Reduced	Strongly reduced	Significant colocalization within RCLS	Significant MT cage reduction and MT colocalization within RCLS	pPERK: similar to WT<br>CHOP: significantly high in nucleus and RCLS
D290N (Walker B – ATP hydrolysis)	RCLS	Significantly less compact	Significantly Reduced	Strongly reduced	Significant colocalization within RCLS	Significant MT cage reduction and MT colocalization within RCLS	pPERK: similar to WT<br>CHOP: significantly high in nucleus and RCLS

R461Q (RNA-binding mutant)	RCLS	Similar to WT	Similar to WT	Strongly Reduced	Significant colocalization within RCLS	Significant MT cage reduction and MT colocalization within RCLS	pPERK: similar to WT<br>CHOP: significantly high in nucleus and RCLS

ZIKV-MR766 (MR-ZIKV); Wild-type (WT); Replication compartment (RC); Replication compartment-like structure (RCLS); Endoplasmic reticulum (ER), Microtubule (MT); Not-significant (ns); Not applicable (N/A); Non-structural (NS), Thapsigargin (TG).
